# The evolving landscape of IL-10, IL-22 and IL-26 in pleurisy especially in tuberculous pleurisy

**DOI:** 10.1186/s12931-024-02896-x

**Published:** 2024-07-13

**Authors:** Qian Niu, Meng Wang, Xian-Sheng Liu

**Affiliations:** 1 Department of Respiratory and Critical Care Medicine, Shanxi Bethune Hospital, Shanxi Academy of Medical Sciences, Tongji Shanxi Hospital, Third Hospital of Shanxi Medical University, Taiyuan, 030032 China; 2 Department of Respiratory and Critical Care Medicine, Tongji Hospital, Tongji Medical College, Huazhong University of Science and Technology, Wuhan, 430030 China; 3 Department of Pathophysiology, School of Basic Medicine, Tongji Medical College, Huazhong University of Science and Technology, Wuhan, 430030 China; 4 Department of Pathology, Baoji Gaoxin Hospital, Baoji, 721000 China

**Keywords:** IL-10, IL-22, IL-26, Pleurisy

## Abstract

Pleurisy can be categorized as primary or secondary, arising from immunological, tumorous, or microbial conditions. It often results in lung structure damage and the development of various respiratory issues. Among the different types, tuberculous pleurisy has emerged as a prominent focus for both clinical and scientific investigations. The IL-10 family, known for its anti-inflammatory properties in the human immune system, is increasingly being studied for its involvement in the pathogenesis of pleurisy. This review aims to present a detailed overview of the intricate role of IL-10 family members (specifically IL-10, IL-22, and IL-26) in human and animal pleuritic diseases or relevant animal models. These insights could serve as valuable guidance and references for further studies on pleurisy and potential therapeutic strategies.

## Introduction

Pleurisy is an inflammatory lesion that occurs in the pleura, characterized by chest pain and pleural effusion. It can be caused by various internal and external factors, primarily involving immunological, tumorous or microbial diseases, which can damage lung structure and lead to respiratory issues [1, 2]. The pleura is divided into visceral pleura and parietal pleura based on attachment location. The space between them forms the pleura cavity with negative pressure, lined with mesothelium and containing resident macrophages, mast cells and lymphocytes [3, 4]. In the event of inflammation in pleura microenvironment, pleural mesothelial cells (PMCs) release chemokines, such as IL-8, MIP-1α, and MCP-1, attracting neutrophils and mononuclear cells to the pleural space [5–8]. This leads to a series of interactions between PMCs and inflammatory or immune cells, resulting in pathophysiological changes. Tuberculous pleurisy (TP), a common form of pleurisy, is often caused by mycobacterium tuberculosis (MTB) infection in the pleura. The pathophysiological process of TP involves of the accumulation of immune cells, increased pleural vascular permeability, and protein-rich fluid buildup, reflecting a strong delayed-type hypersensitivity response to MTB. T lymphocytes, especially CD4^+^T cells, are heavily present in the pleural cavity to combat MTB [9–13].

The IL-10 family, consisting of IL-10, IL-19, IL-20, IL-22, IL-24, IL-26, IL-28 A, IL-28B, and IL-29 [14], includes potential immune response deactivators such as IL-10 and TGF-β. Studies have shown elevated levels of IL-10 and TGF-β in tuberculosis (TB) patients’ serum and peripheral blood mononuclear cells (PBMCs) [15]. These immunosuppressive cytokines not only suppress T cell activity in response to MTB, but also induce T cell anergy by reducing the cell surface expression of co-stimulatory and antigen-presenting molecules on MTB-infected monocytes [16, 17]. The IL-10 family has been a focus in understanding the mechanism of pleurisy, particularly TP. Research indicates that higher IL-10 levels are linked to increased pleural necrosis, along with elevated levels of TNF and IFN-γ [18]. This review will delve into the role and mechanism of IL-10 family members, particularly IL-10, IL-22, and IL-26 in the development of pleurisy based on recent literature findings.

## Research status of IL-10 and pleurisy

### Research condition of IL-10

IL-10, recognized as a key anti-inflammatory cytokine in the human immune response, is the most extensively studied member of the IL-10 family. It is primarily produced by CD4^+^T helper 2 cells (Th2), monocytes and B cells, existing as a homodimer composed of two tightly packed 160-amino-acid proteins. The biological function of IL-10 is mainly associated with the regulatory mechanisms that govern the magnitude and duration of inflammatory response [19]. IL-10 exerts its anti‑inflammatory effects by inhibiting activation and function of both innate and adaptive immune responses [14, 20]. Its significant impact arises from its specific inhibition of the expression of proinflammatory genes in myeloid cells, particularly targeting the production of cytokines and chemokines. Furthermore, IL-10 effectively suppresses the production of various pro-inflammatory cytokines and chemokines, such as IL-1α, IL-1β, IL-3, IL-6, IL-8, TNF-α, IFN-γ, G-CSF, GM-CSF, and MIP-1α. Moreover, it plays a role in reducing DNA stability, thereby modelating the entire inflammatory process [21–26]. In addition to these functions, IL-10 has various other targets. For instance, it enhances the release of the IL-1 natural receptor antagonist (ILRA), inhibits the release of free oxygen radicals and inducible nitric oxide, prevents the transcriptional factor NF-κB from the cytoplasm to the nucleus, and inhibits the phosphorylation of ERK1/2 [23, 24, 27]. While IL-10 is essential in resolving inflammatory processes and safeguarding inflammatory tissue from damage during acute and chronic infections, excessive or inappropriate production of IL-10 can potentially compromise host defenses, leading to the proliferation or persistence of pathogens [28].

### IL-10 and carrageenan-induced pleurisy

Injection of carrageenan (Car, a high molecular weight sulfated polysaccharide isolated from marine algae) into the pleural space induces pleurisy, characterized by the rapid migration of neutrophils from the bloodstream to the inflamed tissue to aid in tissue breakdown and remodeling [29]. This method is frequently utilized to study the mechanisms of acute inflammation and evaluate the effectiveness of anti-inflammatory drugs [30, 31]. Apart from causing paw edema, carrageenan exposure in the pleural cavity also prompts a local inflammatory response, facilitating the infiltration of polymorphonuclear leukocytes (PMN), neutrophils, and monocytes, as well as the excessive production of neutrophil-derived reactive oxygen species (ROS), such as hydrogen peroxide, superoxide, and hydroxyl radical. This process is accompanied by the release of other neutrophil-derived mediators, which stimulate pleural fluid exudation, lung parenchyma injury, alveolar hemorrhage, interstitial thickening, and ultimately result in pulmonary dysfunction [32–35].

The number of F4/80^+^ cells significantly increases after the mice are injected with Car in pleural cavity [36]. In this model, oxidative stress emerges as a crucial factor that accelerates inflammation by stimulating substantial inflammatory cell infiltration and the release of inflammatory mediators, which worsens lung injury [37]. Neutrophil aggregation and macrophage activation lead to the production of pro-inflammatory cytokines, such as IL-1β, IL-6, IL-17, TNF-α, IFN-γ, iNOS, and COX2, which are closely linked to the severity of pleurisy and lung injury induced by Car [36, 38]. Therefore, oxidative stress and subsequent inflammation are identified as the primary causes of Car-induced pleurisy and lung injury. Some studies have also suggested that Car triggers the inflammatory response by targeting various pathways, including NF-κB, NLRP3, MAPK, and STAT3 pathways, as previously described [32, 37, 38].

While one study found a decrease in IL-10 levels following Car injection [39], most studies indicate that Car injection into the pleural cavity can actually increase IL-10 expression locally or systemically. Murai’s research demonstrated a peak in IL-10 levels in the lavage fluid at the 7th hour post Car pleural injection [40]. IL-10 was found to have a notable inhibitory effect on neutrophil migration and showed strong anti-inflammatory actions, particularly inhibiting leukocyte migration to the pleural cavity. This anti-inflammatory effect led to reduced fluid leakage during the early phase (4 h) of Car-induced inflammation, although not during the later phase (48 h) [41]. The release of IL-10 was partially triggerd by PGE2, with high doses of indomethacin resulting in a decrease in IL-10 levels in exudate fluid post Car injection [42, 43]. This decrease in IL-10 was linked to reductions in IL-17 A levels, exudation degree, and leukocyte migration, ultimately dampening the inflammatory response to Car. However, conflicting reports exist regarding the impact of dexamethasone and indomethacin on IL-10 mRNA and protein levels [44].

Pre-treatment of IL-10^WT^ mice with anti-IL-10 antibody, as well as IL-10^KO^ mice, resulted in a significant increase in pleural exudate, a marked increase of NO and PGE2 in exudate, more severe lung injury, inflammatory cell infiltration, and elevated levels of MIP-1α and MIP-2 along with increased MPO activity. Cuzzocrea and colleagues suggested that IL-10 might regulate COX-2 protein expression and activity following Car injection, leading to enhanced PGE2 formation and contributing to the observed inflammatory process [45].

**Fig. 1:**
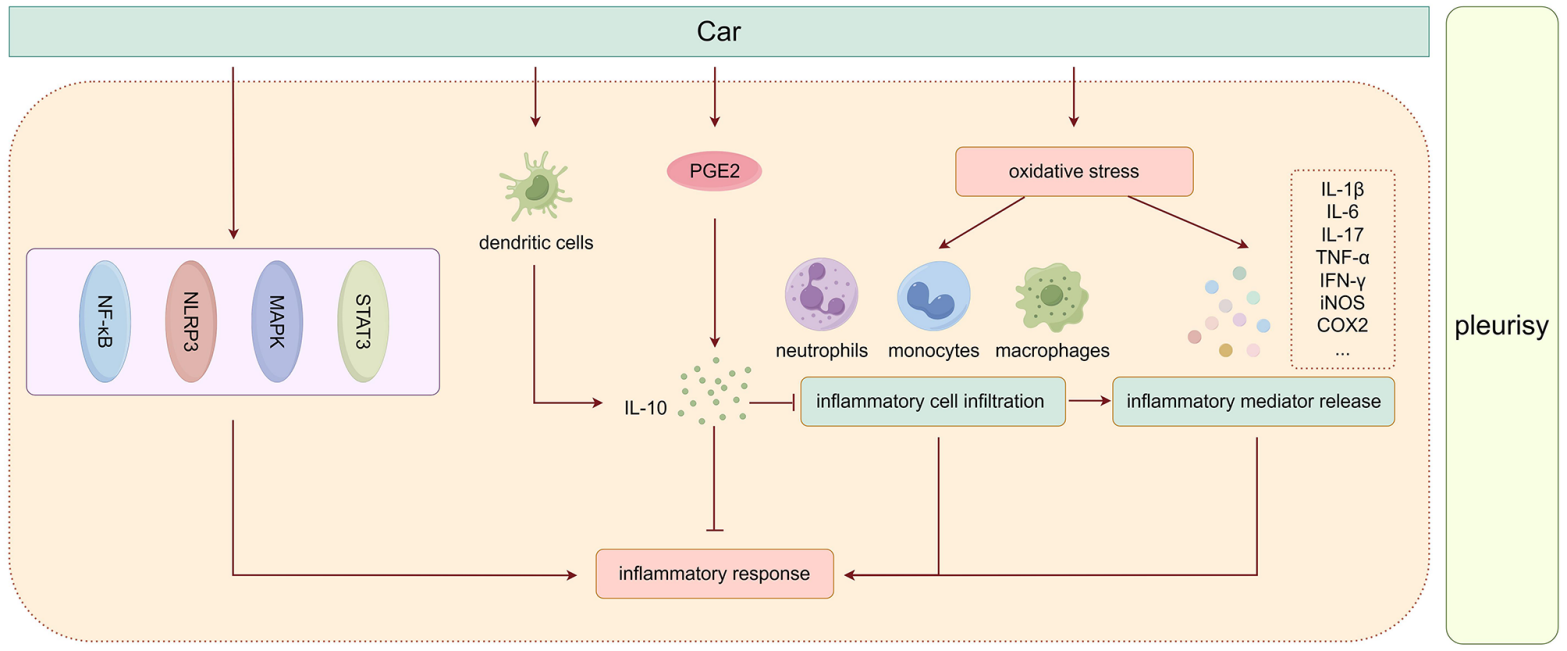
The role of IL-10 in Car-induced pleurisy

The release of IL-10 is not only stimulated by PGE2 but also regulated by other pathways. Car promotes the ex vivo production of IL-12 and IL-10 by dendritic cells and promote their migration to the spleen’s periarteriolar lymphoid sheath area. Car, as a kinin system activator, stimulates B2R (a heterotrimer G protein-coupled receptor)-dependent IL-12 production in vivo. In the presence of ACE inhibition, dendritic cells preferentially produce IL-10 in response to low levels of endogenous kinins. Increased IL-10 production by dendritic cells is observed in response to lysyl-bradykinin (LBK) and captopril. IL-10 counteracts the proinflammatory activity of BK by down-regulating IL-12 production. This suggests that the B2R pathway’s activation of innate immunity is regulated by kinin-degrading peptidases and IL-10-mediated down-regulation [46]. Overall, IL-10 increases reactively in this model to resist Car-induced pleural inflammation. The role of IL-10 in Car-induced pleurisy is illustrated in Fig. 1, based on the literature description.

Car-induced mouse pleurisy provides insights into the mechanisms of pleurisy development and exacerbation, highlighting the role of IL-10. Additionally, this model has been effectively utilized to study the anti-inflammatory effects of various drug therapies in vivo. These therapies, such as non-peptide kinin B(1) receptor antagonists, pioglitazone, and others, have shown promising results in reducing cellular migration, total cell counts, and inhibiting inflammatory activities. The findings from this research serve as a valuable foundation for advancing clinical treatments for pleurisy.

### IL-10 and angiotensin-(1–7)-induced pleurisy

**Fig. 2:**
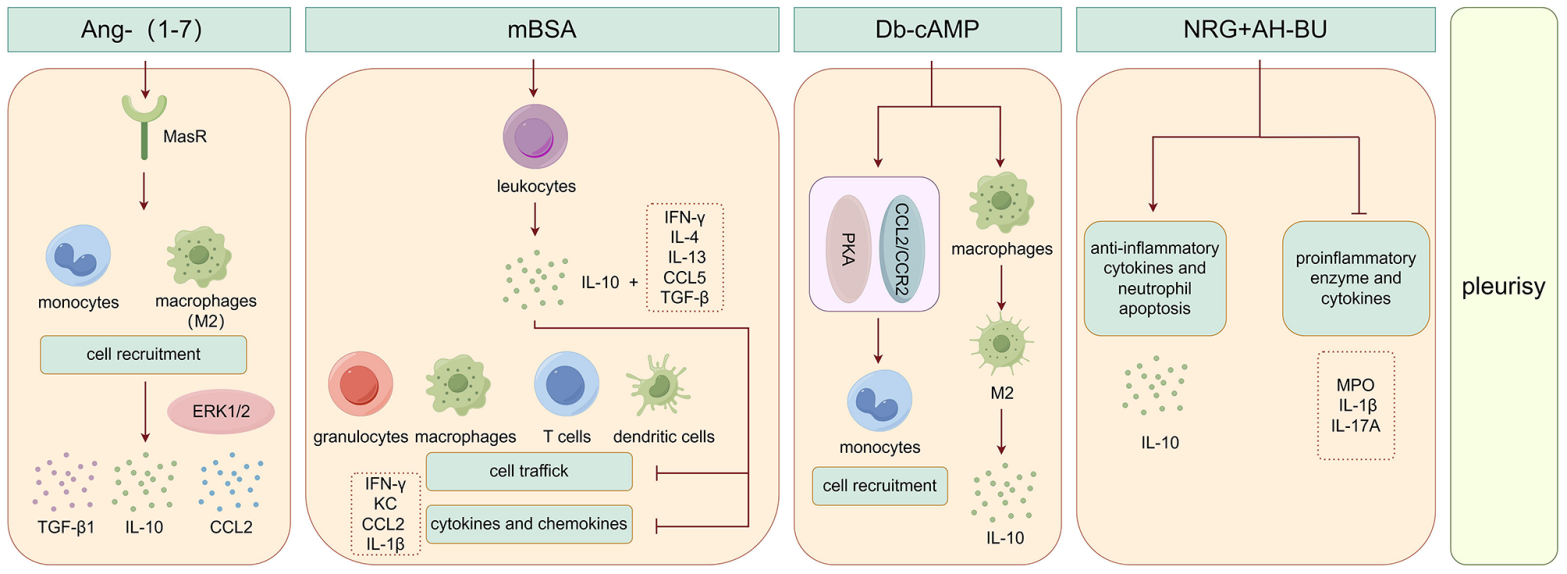
The role of IL-10 in Ang- (1–7)-/mBSA-/db-cAMP-/NRG and AH-BU-induced pleurisy

Angiotensin- (1–7) (Ang- (1–7))-induced pleurisy is associated with increased IL-10 production and recruitment of immune cells into the pleural cavity. Specifically, Ang-(1–7) enhances cell recruitment of monocytes and macrophages into the pleural cavity, leading to increased production of IL-10, TGF-β1 and CCL2. Additionally, Ang-(1–7) boosts IL-10 and TGF-β production in bone marrow derived macrophages’ (BMDMs) in a time-dependent manner, without affecting levels of CXCL1, TNF-α and IL-6, which are proinflammatory cytokines [47]. M2 macrophages, known for secreting anti-inflammatory cytokines like IL-10 and TGF-β, also play a role in this process [48, 49]. The ERK1/2 pathway is suggested to enhance IL-10 production in macrophages [50]. Studies propose that IL-10 production is initially triggered by the Ang-(1–7)-MasR axis, leading to the expression of classical M2 markers in recruited macrophages, such as Arg1 and Ym1. Regulatory cytokines inducted by Ang-(1–7) may promote efferocytosis and polarization of macrophages towards regulatory phenotypes in the tissue [51–53]. Further research on the cellular sources of IL-10 and TGF-β post Ang-(1–7) injection will provide a clearer picture of players involved in the anti-inflammatory and pro-resolving effect of Ang-(1–7). The role of IL-10 in Ang- (1–7)-induced pleurisy is illustrated in Fig. 2.

### IL-10 and methylated BSA-induced pleurisy

Methylated-BSA (mBSA) thoracic injection induces pleurisy and leads to a prolonged increase in IFN-γ, IL-4, IL-10, IL-13, and CCL5 expression in challenged mice. Subsequent observations show a sustained upregulation of IL-4, IL-10, IL-13, and TGF-β mRNA levels by pleural exudate leukocytes, indicating potential immunoregulatory activities. Researchers investigated IL-10’s impact on leukocyte trafficking in the mBSA-induced pleuritis model, revealing a modest effect on granulocytes, macrophages, T cells, and dendritic cells. IL-10 treatment notably reduces IFN-γ levels at 24 h post-challenge and inhibits KC, CCL2, and IL-1β by 25%~35% at 6 h [54]. Further research aims to identify key molecules in this response and clarify the role of IL-10. Ongoing studies are depicted in Fig. 2, illustrating the involvement of IL-10 in mBSA-induced pleurisy.

### IL-10 and db-cAMP-induced pleurisy

Db-cAMP-induced pleurisy results in a non-phlogistic cell recruitment process, with an early (4 h) increase of IL-10 levels persisting for up to 48 h. Injection of db-cAMP into the pleural cavity of mice leads to monocytes recruitment through PKA and CCL2/CCR2 pathways, while not significantly affecting neutrophil numbers or the levels of neutrophil chemoattractant CXCL1, TNF-α and IL-6. Additionally, db-cAMP promotes the transformation of BMDMs into M2 phenotype, as shown by increased expression of Arg-1, CD206, Ym-1 and IL-10 (M2 markers). Db-cAMP also has a synergistic effect with IL-4 in inducing STAT3 phosphorylation [55]. The role of IL-10 in db-cAMP-induced pleurisy is illustrated in Fig. 2.

### IL-10 and noreugenin and α-hydroxy-butein-induced pleurisy

The study illustrated that noreugenin (NRG) and α-hydroxy-butein (AH-BU) displayed significant anti-inflammatory and antioxidant properties. These isolated compounds demonstrated a noteworthy anti-inflammatory impact by inhibiting proinflammatory enzyme (MPO) or cytokines (IL-1β and IL-17 A), while also increasing the levels of anti-inflammatory cytokine (IL-10) and promoting neutrophil apoptosis. Additionally, NRG and AH-BU were found to enhance IL-10 levels in the fluid leakage of the mouse pleural cavity under the same experimental conditions [56]. Figure 2 illustrates the role of IL-10 in NRG and AH-BU-induced pleurisy.

Animal models have elucidated the potential pathogenesis and pathophysiology in vivo. These models can be valuable in assessing the significance of IL-10 in pleurisy, potentially opening up new therapeutic avenues for its treatment.

### IL-10 and tuberculous pleurisy

IL-10 is known to exacerbate tuberculosis by inhibiting the protective arm of innate immune response and antigen-specific cellular immune response. Levels of IL-10 are significantly elevated in tuberculous pleural effusion (TPE) [57, 58]. CD8^+^T cells are crucial in controlling TB by releasing Th1 cytokines like IFN-γ and TNF-α, as well as lysing MTB-infected macrophages and reducing MTB viability [59–61]. Previous research has known that IL-10, produced by macrophages and stimulated by MTB cell wall components in TPE, can suppress the local immune response. Additionally, IL-10 produced by macrophages is specifically concentrated in the pleural space [62, 63].

Recent studies on the intricate cytokine network have indicated that the relative balance between different cytokine levels may offer a more accurate reflection of their overall impact on the immune response compared to their individual concentrations. Maintaining a proper equilibrium between TNF-α and IL-10 is crucial for regulating or preventing the spread of MTB [64]. IL-10 has been shown to inhibit the production of IL-1β and TNF-α by pleural macrophages when induced by LPS [65]. The ratio of INF-γ/IL-l0 is significantly higher in TPE compared to malignant or other types of effusions [66], highlighting the importance of pro- and anti-inflammatory cytokines [67]. Elevated levels of IFN-γ in TPE indicate a heightened immune response. IL-10 produced by T cell counteracts the function of macrophage in MTB infection by counteracting the effect of IFN-γ and suppressing TNF-α and NO released by MTB-infected macrophages, thereby potentially preventing severe inflammation and tissue damage [68, 69].

IL-10 increases the expression of the M2c markers CD16 (Fc γ RIII) and CD163, which are known to help prevent tissue inflammation. This underscores the importance of macrophage polarization in influencing T cell cytotoxic response during MTB infection [70]. This discovery is particularly relevant in context of TB infection, where the elevated levels of IL-10 could alter macrophage susceptibility to lysis by CD8^+^T cell effectors at the infection site [71]. IL-10 not only hinders T cells activity in response to MTB, but also induces T cells anergy by reducing the expression of costimulatory and antigen-presenting molecules on MTB-infected monocytes [16, 17, 72].

Treg plays a crucial role in regulating the immune response in pleural effusions by controlling the activity of CD4^+^ and CD8^+^T cells and their production of IFN-γ. Treg cells efficiently inhibit the production of IFN-γ in CD4^+^ and CD8^+^T cells triggered by MTB, impacting their ability to response to MTB even in a Th1 microenvironment observed in TPE [73]. Generally, Treg cells are closely linked to the local immune response in TB patients, with their interaction with Teff cells influencing the type of immune response and clinical presentation of the disease. Studies have shown an increase in Treg cell numbers in TP patients, with Treg cells suppressing Teff cell function through IL-10 secretion [73, 74]. Blocking the effect of IL-10 with monoclonal antibodies may restore effector cell function, aiding in disease control [75]. Further research is needed to explore the factors contributing to the imbalance between Treg and Teff cells and the specific recruitment of T cells at the pathological site.

Th9 cells are generated from naïve CD4^+^T precursors through the influence of both TGF-β and IL-4. These cells are known for their production of IL-9 and IL-10. In cases of TPE, Th9 cells have been identified in higher quantities compared to blood samples from the same individuals [76–78].

The research indicates that activated B cells, specifically TPE-B cells, may have a significant impact on the generation of IL-10, a cytokine known for its immunoregulatory functions [79–82]. These specific B cells have been observed to modulate the immune response of CD19^−^cells in TPE through an IL-10-dependent mechanism. The secretion of IL-10 by TPE-B cells is thought to control the production of IFN-γ by T cells and NK cells. It’s hypothesized that IL-10 generated by TPE-B cells could hinder IFN-γ production by TPE-NK cells through two possible mechanisms: direct signaling via IL-10R signaling and indirect suppression of IL-12 production by antigen-presenting cells in TPE [67].

**Fig. 3:**
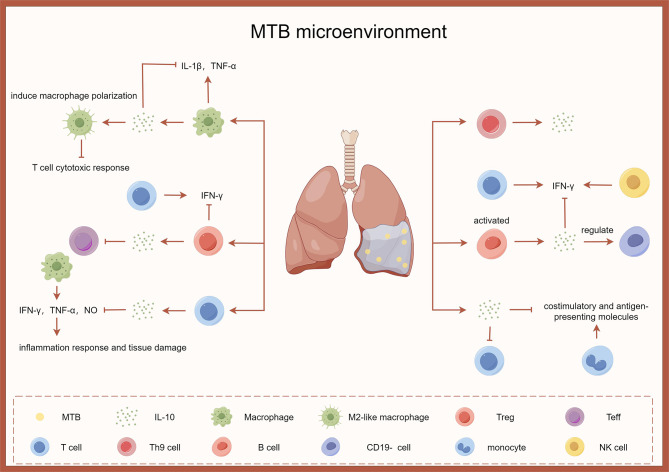
The role of IL-10 in tuberculous pleurisy

The exsiting research indicates that regardless of the specific immune cell type producing IL-10 in the microenvironment of tuberculous pleurisy, it plays a crucial role in dampening inflammation. The literature highlights the participation of IL-10 in context of tuberculous pleurisy in Fig. 3.

Infections by M.tuberculosis in the pleural cavity remain a significant global health concern. Genetic studies have unequivocally shown that cell-mediated mechanisms of host defense, involving innate immunity and T cells, play a crucial role in controlling tuberculous pleurisy. Despite extensive research efforts, the development of effective therapies for tuberculous pleurisy has proven to be largely unsuccessful. The data presented in this study highlight the various immune sources of IL-10 during M.tuberculosis infection, providing compelling evidence for compartmentalization of Th1 cytokines and IL-10 at the site of disease in individuals with a resilient immune response to mycobacterial infection. Furthermore, these findings suggest that IL-10 contributes to persistence of mycobacterial infections within host macrophages. For instance, experiments involving transgenic mice that secrete IL-10 from the T cell compartment and subsequent infection with Calmette-Guérin bacillus (Mycobacterium bovis) indicate that the excess IL-10 has minimal impact on T cell function or development during the immune response to the pathogen. The application of tuberculous pleurisy mouse models will aid in identifying its underlying mechanisms and the studying the regulatory role of IL-10 in this disease.

### IL-10 and malignant pleurisy

Previous studies have shown a notable increase in IL-10 levels in malignant pleural effusion (MPE) when compared to peripheral blood [83]. The average IL-10 value in MPE caused by lung cancer is lower than that in TPE, but tends to be higher than that in transudative pleural effusion [65]. Both effusion and blood IL-10 levels are notablely higher in non-adenocarcinoma patients compared to adenocarcinoma patients [84]. Male lung cancer patients have higher blood IL-10 levels than females [84]. Furthermore, IL-10 may have a key role in tumor-induced immunosuppression [85].

**Fig. 4:**
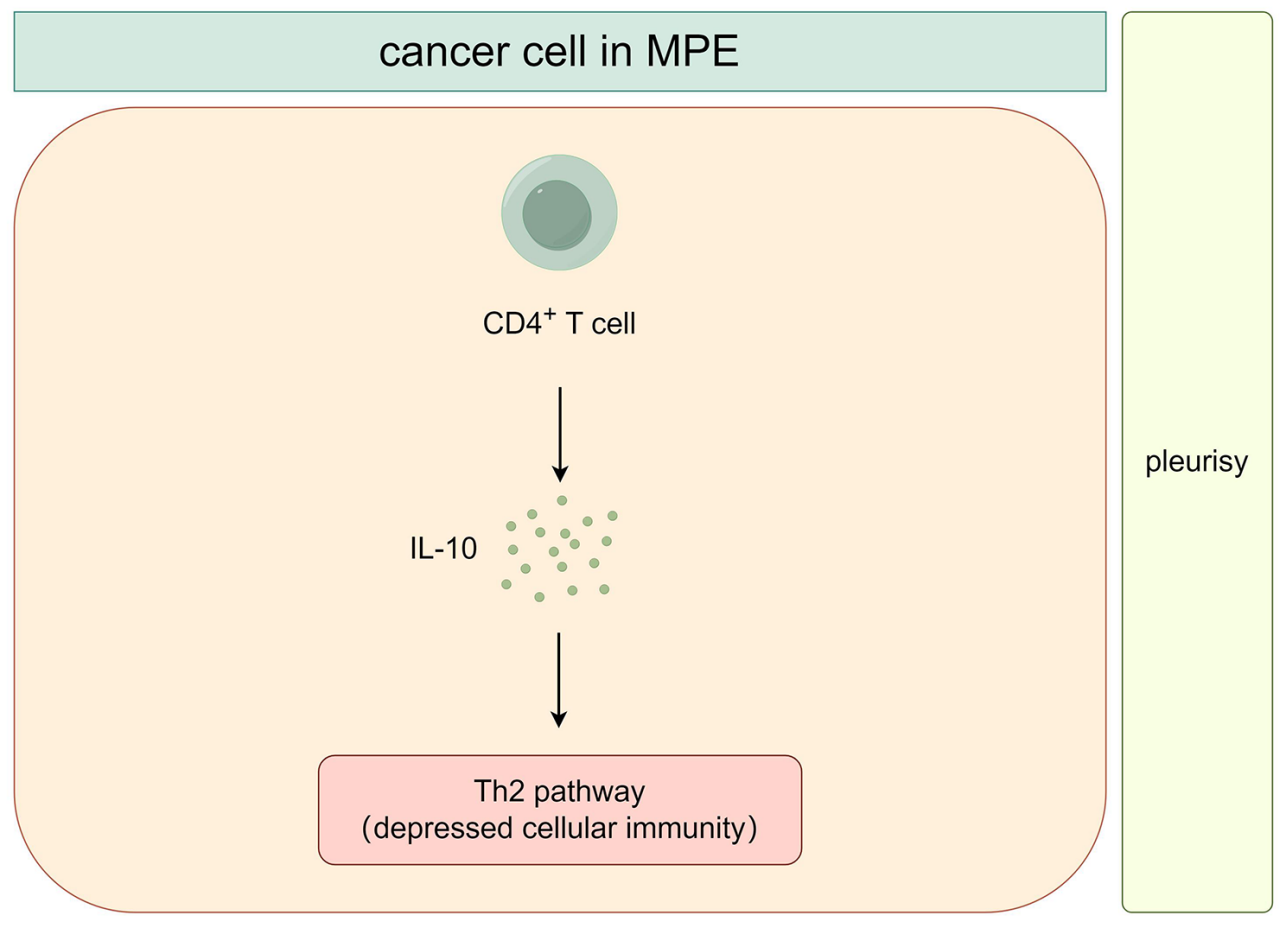
The role of IL-10 in malignant pleurisy

Pleural effusions from lung cancer often show significantly elevated levels of CD4^+^T lymphocytes, which are the primary source of IL-10 production [86]. TPE tends to have higher levels of both IFN-γ and IL-10, indicating an active local cellular immune reaction that favors the Th1 pathway (enhanced cellular immunity). Conversely, MPE may exhibit dominance towards the Th2 pathway [86, 87], with high levels of IL-10 with low or undetectable IFN-γ expression, suggesting a depressed cellular immunity [84]. In summary, TPE shows a more active local cellular reaction compared to MPE, with lung cancer patients with a MPE predominantly displaying a Th2 pathway and an immunosuppressed state. Based on the literature description, Fig. 4 illustrates the role of IL-10 in malignant pleurisy.

The presence of IL-10 suggests the potential for a universal assay to track the progression and recurrence of malignant pleurisy. It could serve as a dependable marker in pleural or plasma samples and could also support further investigation into using checkpoint blockade as a new adjuvant therapy for malignant pleurisy.

## Research status of IL-22 and pleurisy

### Research condition of IL-22

IL-22, a member of the IL-10 cytokine family, exhibits diverse biological functions and is produced by various lymphocytes such as Th17, Th22, NK, γδT lymphoid tissue inducer (LTi) and LTi-like cells [88]. The IL-22 receptor complex, composed of IL-22R1 and IL-10R2, is predominantly found on non-hematopoietic epithelial and fibroblast cells in multiple tissues [89–92]. Depending on the context, IL-22 can have both protective and pathological effects. Its signaling occurs through the IL-22/IL-22R complex and involves pathways like Jak1, Tyk2, STAT1, STAT3, and STAT5 [92, 93]. Elevated IL-22 levels are associated with T cell-mediated inflammatory conditions like psoriasis, Crohn’s disease and rheumatoid arthritis [92, 94–96]. IL-22 plays a role in immunity by stimulating antimicrobial peptides, chemokines for neutrophil-recruiting, mucins, and acute phase proteins, crucial for tissue integrity during chronic inflammation. Additionally, IL-22 is implicated in the pathogenesis of certain disease and is a potential therapeutic target [97–99]. As research progresses on IL-10 family, it is essential to understand the distinct roles and mechanism of each member of developing effective therapeutic strategies.

### IL-22 and tuberculous pleurisy

Limited research exists on the role of IL-22 in non-tuberculous pleurisy. A study investigated the expression of IL-22 mRNA by PFMC after exposure to immune-dominant peptides of 6 kDa early secretory antigenic target (ESAT-6), culture filtrate pritein-10 (CFP-10) or with BCG in vitro. Results showed that these stimuli led to increased levels of IL-22 mRNA transcription and protein production compared to control cultures [100]. IL-22 was detected in TPE and was found to be higher than in the corresponding blood samples, indicating a potential role in TPE pathogenesis [101]. The synergistic effect of IL-1β with IL-6, IL-23, and TGF-β was shown to promote Th17 differentiation and sustain IL-22 production in effector cells [102, 103]. Interestingly, IL-22 can also be produced by non-Th17 cells independently of IL-17. In experimental MTB infection, Th1 and Th22 cells were identified as the main sources of IL-22, rather than Th17 cells [104, 105]. Th22 cells, similar to Th1 and Th17 subsets, retain membrane-bound IL-22 for efficient cell-cell communication during anti-MTB immune responses [106]. Th22 cells have also been observed in TPE [100, 107]. Studies have demonstrated that the numbers of Th22, Th17, and Th1 cells in TPE are significantly higher compared to blood samples [108].

IL-22 is one of the few cytokines that exist in both secretory and membrane-bound forms. Membrane-bound IL-22 has a prolonged half-life and remains present even in a TB inflammatory environment. IL-22 can be intracellular, secreted by T cells, or expressed on the membranes of T cells [109]. Significant expression of IL-22R is observed on PMCs isolated from TPE [108]. PMCs have the ability to induce differentiation of Th22, Th17, and Th1 cells from naïve CD4^+^T cells though antigen presentation. The majority of IL-22-producing T cells in the pleural fluid are CD4^+^T cells, with a lesser presence of IL-22 within CD8^+^T cells. IL-22 can enhance the proliferation response of CD4^+^T cells induced by antigen presentation by PMCs. In macaques infected with MTB, CD4^+^T cells can develop into T effector cells expressing membrane-bound IL-22 after de novo IL-22 production. Importantly, membrane-bound IL-22^+^T cells can be detected in MTB-infected macaques and have been shown to inhibit intracellular MTB replication in macrophages [106, 108]. As early as 16 h post-injury, both IL-22 and IL-17 exhibit significant and persistent effects on wound closure mediated by PMC layer regeneration. It is possible that antigen-specific IL-22-producing T cells are recruited to affected tissues through chemokines released by infected resident macrophages and dendritic cells [109]. Similar to its counterpart IL-17, IL-22 contributes to controlling extracellular bacterial infection [110]. A study by Volpe and colleagues demonstrated that the regulation of human IL-17 and IL-22 production varies during cytokine-induced Th cell differentiation, suggesting a differential involvement of IL-22 in Th1 cell-mediated immune responses [111].

The presence of PMCs in the microenvironment of TP is associated with the promotion of epithelial function. This interstitial transition process is primarily driven by the abundance of inflammatory cytokines found in the pleural effusion. In TPE, IL-22 effectively activates STAT3 serine at position 727 (Ser727). Through activation of the STAT3 pathway, IL-22 can counteract the basal level of PMC in TP and prevent PMC epithelial transformation induced by IFN-γ, thereby potentially exerting a protective role against pleural fibrosis in TP [112].

**Fig. 5:**
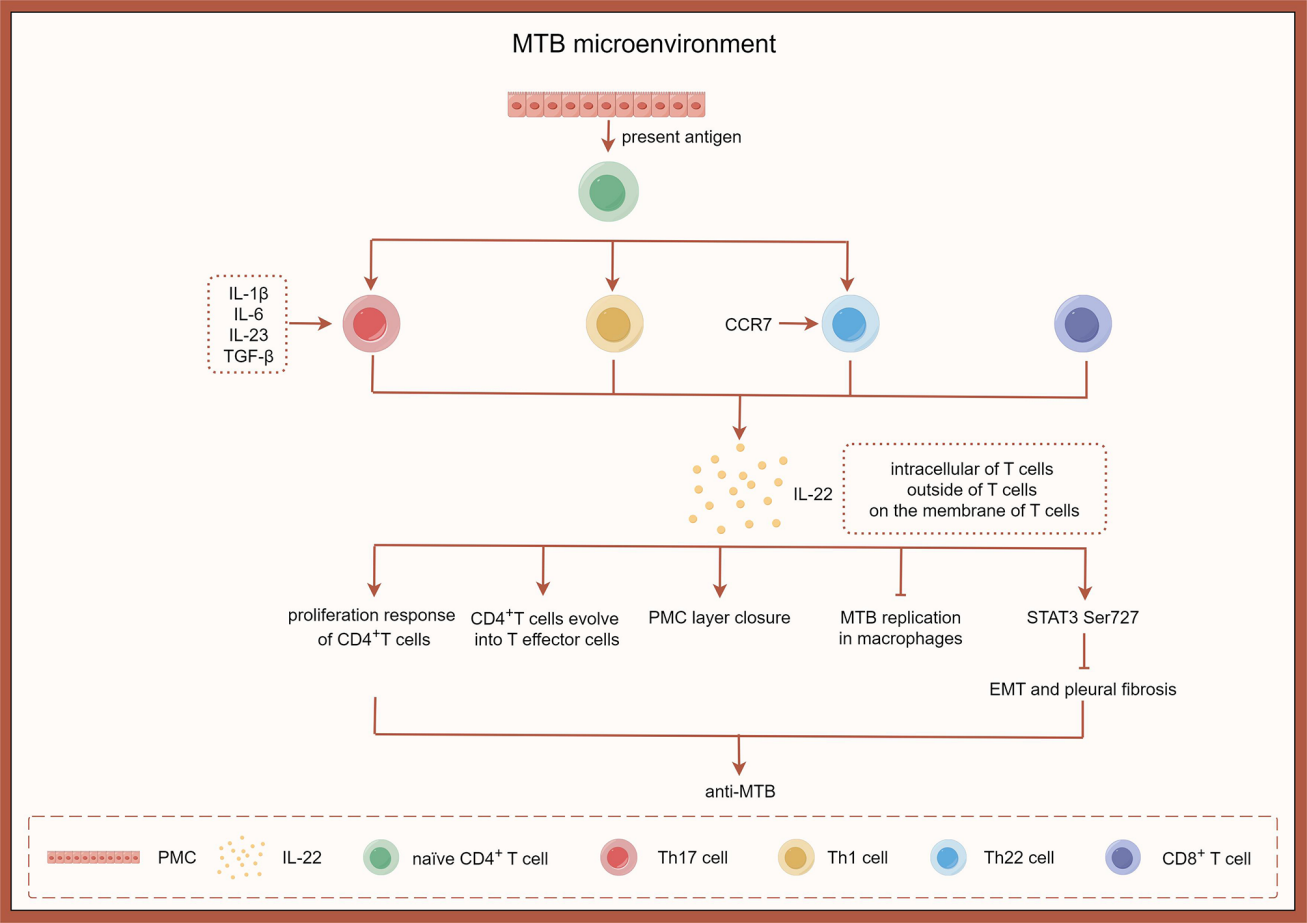
The role of IL-22 in tuberculous pleurisy

Central memory cells are durable populations capable of extensive expansion for an effective secondary immune response, potentially providing long-lasting protection against TB [100]. The presence of CCR7 expression confers lymph node homing potential to antigen-experienced CD45RA^−^T cells, characteristic of central memory cells, while the absence of CCR7 allows migration to the site of infection, typical of effector cells [113]. Interestingly, Th22 cells in the TPE environment exhibit significant up-regulation of CCR7 expression [108]. Ye and colleagues observed that most Th22 cells expressed high levels of CD45RO in both TPE and blood samples, with low levels of CD45RA and CD62L, indicating their status as effector memory cells, especially those present in TPE [108]. Additionally, pleural Th22 cells demonstrate moderate levels of CCR7 expression compared to blood Th22 cells, facilitating easy migration into the pleural space during MTB infection [108]. Collectively, IL-22- or IL-17-producing CD4^+^T cells found in pleural fluid represent central memory cell populations that may contribute to long-lasting protection against MTB infection in TP patients [108]. Therefore, it is believed that IL-22 mediates a protective immune response against MTB. Based on literature descriptions, we have depicted the role of IL-22 in tuberculous pleurisy in Fig. 5.

## Research status of IL-26 and pleurisy

### Research condition of IL-26

IL-26 is a 171-amino acid protein that belongs to the IL-10 family [114]. It shares a common second receptor chain (IL-10R2) with IL-22, forming active receptor complexes and initiating signaling [115]. Primary T cells, NK cells, and T cell clones can produce IL-26 upon stimulation with a specific antigen [116]. Previous studies have shown that human Th1 clones co-express IL-26 along with IFN-γ and IL-22. Th17 cells also co-express it with IL-17 and IL-22 [95, 116–118]. Accumulating evidence suggests that IL-26 plays a proinflammatory role in antibacterial host defense. It enhances neutrophil chemotaxis and directs their mobilization toward infection site [119–121]. Furthermore, elevated levels of IL-26 have been observed in Crohn’s disease and rheumatoid arthritis, indicating its potential involvement as a pathogenic factor in these chronic inflammatory disorders [122–124]. These findings highlight the emergence of IL-26 as an upstream proinflammatory cytokine in the inflammation cascade. Therefore, it holds promise as a therapeutic target for chronic inflammatory disorders. Additionally, there is speculation that IL-26 may negatively affect antimycobacterial activity and could be considered as a candidate gene for tuberculosis susceptibility [125].

### IL-26 and tuberculous pleurisy

**Fig. 6:**
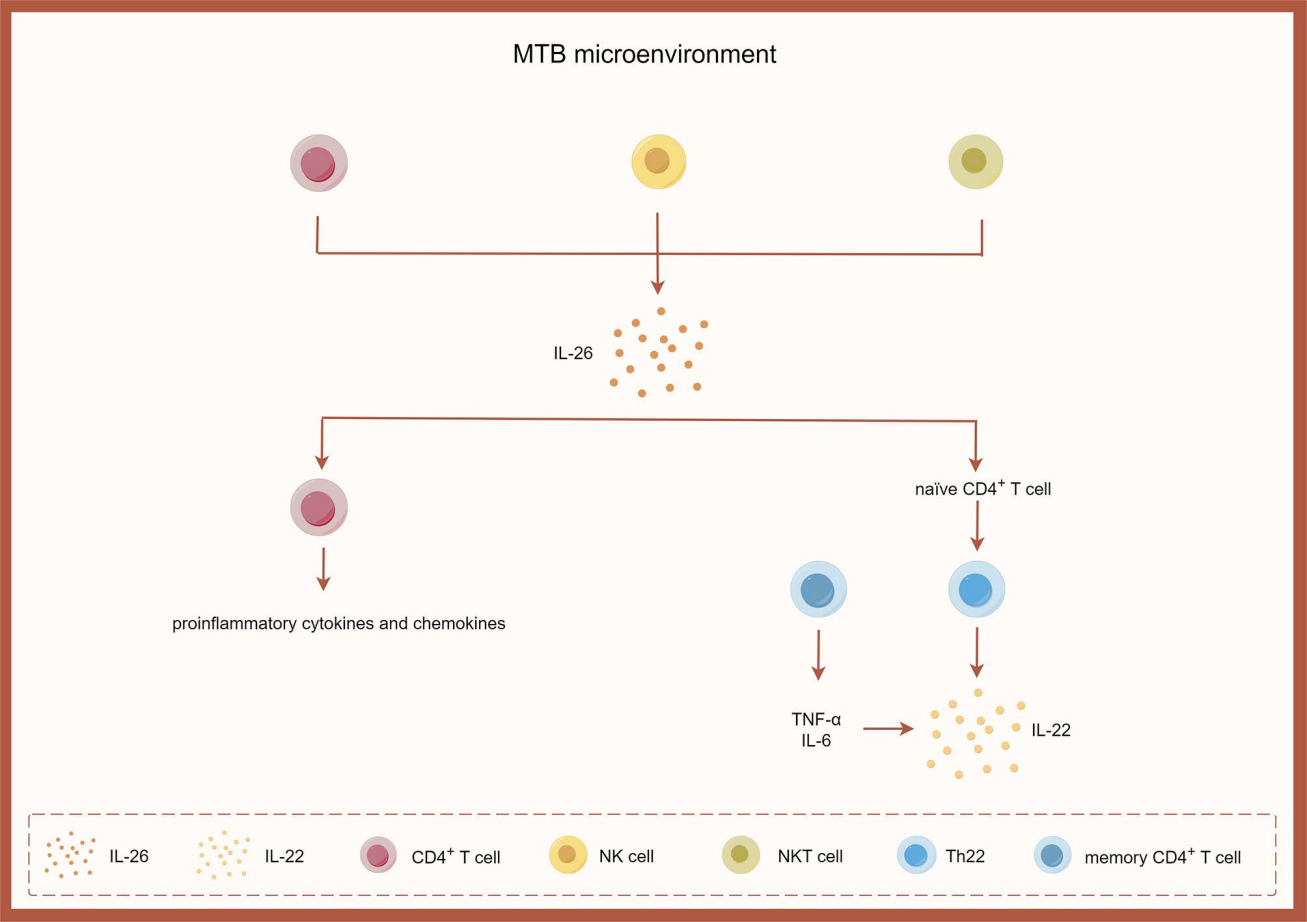
The role of IL-26 in tuberculous pleurisy

TPE is confined to the pleural cavity, which contains protein-rich fluid and numerous immunocompetent cells, particularly CD4^+^T cells [11]. Previous studies have widely demonstrated that CD4^+^T cells are a dominant population in TPE and a major source of IL-26 in the local pleural microenvironment [11, 57]. Most available animal models depend on mice or rats; however neither mice nor rats possess the IL-26 gene, preventing the role of IL-26 in animal models of pleurisy. Additionally, there is no available literature regarding IL-26 in non-tuberculous pleurisy. To date, only one study has investigated the role of IL-26 in tuberculous pleurisy [57], finding significantly higher expression of IL-26 in CD4^+^T cells, NK cells, and NKT cells, but not CD8^+^T cells, in TPE compared to blood samples. A positive correlation was observed between IL-26 levels and the concentrations of proinflammatory cytokines such as IL-8, TNF-α, LDH and ADA in TPE. Moreover, IL-26 concentrations were positively correlated with numbers of pleural lymphocytes, and CD4^+^IL-26^+^ cells were positively correlated with the number of Th1, Th17 and Th22 cells. RT-PCR analysis showed 42-fold higher expression levels of IL-26 in monocytes isolated from TPE compared to corresponding serum samples. Furthermore, IL-26 concentrations were much higher in sera from TPE patients than in those from malignant, infectious, or normal groups. Immunofluorescence analysis revealed strong patterns for IL-10R2 and IL-20R1 (IL-26 receptors) in the parietal pleura isolated from TPE. Double immunofluorescence staining also revealed significant co-expression patterns for both markers on CD4^+^T lymphocytes among mononuclear cells isolated from TPE. Pleural secretion of IL-26 was induced by tuberculosis-specific antigen stimulation via activation by CD4^+^T cells, introducing positive feedback loops with other immune cell types through induction pathways involving proinflammatory cytokine production by activated immune cell subpopulations (e.g., CCL20, CCL22, CCL27). The mRNA encoding CCL20, CCL22, and CCL27 by CD4^+^T cells was also increased in response to IL-26 stimulation. IL-26 facilitated the differentiation of Th22 cells from naïve CD4^+^T cells and augmented the frequency of IL-22-producing CD4^+^T cells primarily through the induction of TNF-α and IL-6 by memory CD4^+^T cells in human TPE. These findings suggest that IL-26 may exert a similar role as IL-22 in combating MTB infection within the pleural cavity. Based on relevant literature, Fig. 6 depicts the involvement of IL-26 in tuberculous pleurisy.

## Conclusions

The body’s defense system maintains a dynamic and evolving balance between pro-inflammatory and anti-inflammatory cytokines, and disruption of this delicate equilibrium can lead to pathological changes. Members of the IL-10 family, including IL-10, IL-22, and IL-26, play crucial roles in regulating inflammatory and immune responses in pleurisy and related animal models through an intricate network of pathways. The actions of the IL-10 family are influenced by variety factors such as timing of cytokines release, site of action, receptor density, presence of competitive or synergistic factors, and tissue responsiveness to each cytokine. It is widely accepted that IL-10, IL-22 and IL-26 inhibit the pathological progression of tuberculous pleurisy by suppressing inflammatory responses while promoting local immune responses. However, the roles and mechanisms of other cytokine within the IL-10 family remain unclear and require further investigation. Identifying differences in the relative abundance or ratios of the IL-10 family members among individual patients with pleurisy could potentially have prognostic value and be associated with different stages of disease.

## Data Availability

No datasets were generated or analysed during the current study.

## References

[1] Ryu S, Fu W, Petri MA (2017). Associates and predictors of pleurisy or pericarditis in SLE. Lupus Sci Med.

[2] Hunter MP, Regunath H. Pleurisy. StatPearls. Treasure Island (FL): StatPearls Publishing Copyright © 2022. StatPearls Publishing LLC.; 2022.

[3] Kroegel C, Antony VB (1997). Immunobiology of pleural inflammation: potential implications for pathogenesis, diagnosis and therapy. Eur Respir J.

[4] Antony VB (2003). Immunological mechanisms in pleural disease. Eur Respir J.

[5] Antony VB, Hott JW, Kunkel SL, Godbey SW, Burdick MD, Strieter RM (1995). Pleural mesothelial cell expression of C-C (monocyte chemotactic peptide) and C-X-C (interleukin 8) chemokines. Am J Respir Cell Mol Biol.

[6] Mohammed KA, Nasreen N, Ward MJ, Mubarak KK, Rodriguez-Panadero F, Antony VB (1998). Mycobacterium-mediated chemokine expression in pleural mesothelial cells: role of C-C chemokines in tuberculous pleurisy. J Infect Dis.

[7] Mohammed KA, Nasreen N, Ward MJ, Antony VB (1998). Macrophage inflammatory protein-1alpha C-C chemokine in parapneumonic pleural effusions. J Lab Clin Med.

[8] Mohammed KA, Nasreen N, Ward MJ, Antony VB (1999). Helper T cell type 1 and 2 cytokines regulate C-C chemokine expression in mouse pleural mesothelial cells. Am J Respir Crit Care Med.

[9] Yao S, Huang D, Chen CY, Halliday L, Wang RC, Chen ZW (2014). CD4 + T cells contain early extrapulmonary tuberculosis (TB) dissemination and rapid TB progression and sustain multieffector functions of CD8 + T and CD3- lymphocytes: mechanisms of CD4 + T cell immunity. J Immunol.

[10] Porcel JM (2009). Tuberculous pleural effusion. Lung.

[11] Light RW (2002). Clinical practice. Pleural effusion. N Engl J Med.

[12] Ferrer J (1997). Pleural tuberculosis. Eur Respir J.

[13] Khalil RY, Khalil MM (1997). Flow cytometric study of T-cell subsets in lymphocytic pleural effusions. Cytometry.

[14] Ouyang W, Rutz S, Crellin NK, Valdez PA, Hymowitz SG (2011). Regulation and functions of the IL-10 family of cytokines in inflammation and disease. Annu Rev Immunol.

[15] Li Q, Li L, Liu Y, Fu X, Qiao D, Wang H (2011). Pleural fluid from tuberculous pleurisy inhibits the functions of T cells and the differentiation of Th1 cells via immunosuppressive factors. Cell Mol Immunol.

[16] Bonecini-Almeida MG, Ho JL, Boéchat N, Huard RC, Chitale S, Doo H (2004). Down-modulation of lung immune responses by interleukin-10 and transforming growth factor beta (TGF-beta) and analysis of TGF-beta receptors I and II in active tuberculosis. Infect Immun.

[17] Zeller JC, Panoskaltsis-Mortari A, Murphy WJ, Ruscetti FW, Narula S, Roncarolo MG (1999). Induction of CD4 + T cell alloantigen-specific hyporesponsiveness by IL-10 and TGF-beta. J Immunol.

[18] Barbosa T, Arruda S, Chalhoub M, Oliveira F, Melo JF, Fidelis R (2006). Correlation between interleukin-10 and in situ necrosis and fibrosis suggests a role for interleukin-10 in the resolution of the granulomatous response of tuberculous pleurisy patients. Microbes Infect.

[19] da Silva AO, Damaceno Alves A, Almeida DA, Balogun SO, de Oliveira RG, Aires Aguiar A (2014). Evaluation of anti-inflammatory and mechanism of action of extract of Macrosiphonia Longiflora (Desf.) Müll. Arg. J Ethnopharmacol.

[20] Saraiva M, O’Garra A (2010). The regulation of IL-10 production by immune cells. Nat Rev Immunol.

[21] Kwilasz AJ, Grace PM, Serbedzija P, Maier SF, Watkins LR (2015). The therapeutic potential of interleukin-10 in neuroimmune diseases. Neuropharmacology.

[22] Bazzoni F, Tamassia N, Rossato M, Cassatella MA (2010). Understanding the molecular mechanisms of the multifaceted IL-10-mediated anti-inflammatory response: lessons from neutrophils. Eur J Immunol.

[23] Locati M, Mantovani A, Sica A (2013). Macrophage activation and polarization as an adaptive component of innate immunity. Adv Immunol.

[24] Mollazadeh H, Cicero AFG, Blesso CN, Pirro M, Majeed M, Sahebkar A (2019). Immune modulation by curcumin: the role of interleukin-10. Crit Rev Food Sci Nutr.

[25] Jung M, Ma Y, Iyer RP, DeLeon-Pennell KY, Yabluchanskiy A, Garrett MR (2017). IL-10 improves cardiac remodeling after myocardial infarction by stimulating M2 macrophage polarization and fibroblast activation. Basic Res Cardiol.

[26] Mahon OR, Browe DC, Gonzalez-Fernandez T, Pitacco P, Whelan IT, Von Euw S (2020). Nano-particle mediated M2 macrophage polarization enhances bone formation and MSC osteogenesis in an IL-10 dependent manner. Biomaterials.

[27] Hovsepian E, Penas F, Siffo S, Mirkin GA, Goren NB (2013). IL-10 inhibits the NF-κB and ERK/MAPK-mediated production of pro-inflammatory mediators by up-regulation of SOCS-3 in Trypanosoma Cruzi-infected cardiomyocytes. PLoS ONE.

[28] Jusek G, Reim D, Tsujikawa K, Holzmann B (2012). Deficiency of the CGRP receptor component RAMP1 attenuates immunosuppression during the early phase of septic peritonitis. Immunobiology.

[29] Nantel F, Denis D, Gordon R, Northey A, Cirino M, Metters KM (1999). Distribution and regulation of cyclooxygenase-2 in carrageenan-induced inflammation. Br J Pharmacol.

[30] Costa R, Fernandes ES, Menezes-de-Lima O, Campos MM, Calixto JB (2006). Effect of novel selective non-peptide kinin B(1) receptor antagonists on mouse pleurisy induced by carrageenan. Peptides.

[31] Fröde TS, Buss Zda S, dos Reis GO, Medeiros YS (2009). Evidence of anti-inflammatory effects of pioglitazone in the murine pleurisy model induced by carrageenan. Int Immunopharmacol.

[32] Gao Y, Lv X, Yang H, Peng L, Ci X (2020). Isoliquiritigenin exerts antioxidative and anti-inflammatory effects via activating the KEAP-1/Nrf2 pathway and inhibiting the NF-κB and NLRP3 pathways in carrageenan-induced pleurisy. Food Funct.

[33] Ward PA (2010). Oxidative stress: acute and progressive lung injury. Ann N Y Acad Sci.

[34] Caiazzo E, Morello S, Carnuccio R, Ialenti A, Cicala C (2019). The Ecto-5’-Nucleotidase/CD73 inhibitor, α,β-Methylene Adenosine 5’-Diphosphate, exacerbates Carrageenan-Induced Pleurisy in Rat. Front Pharmacol.

[35] Lentsch AB, Ward PA (2001). Regulation of experimental lung inflammation. Respir Physiol.

[36] Hou T, Yang M, Yan K, Fan X, Ci X, Peng L (2022). Amentoflavone ameliorates Carrageenan-Induced Pleurisy and Lung Injury by inhibiting the NF-κB/STAT3 pathways via Nrf2 activation. Front Pharmacol.

[37] Ahmad SF, Attia SM, Bakheet SA, Zoheir KM, Ansari MA, Korashy HM (2015). Naringin attenuates the development of carrageenan-induced acute lung inflammation through inhibition of NF-κb, STAT3 and pro-inflammatory mediators and enhancement of IκBα and anti-inflammatory cytokines. Inflammation.

[38] Chedid P, Boussetta T, Dang PM, Belambri SA, Marzaioli V, Fasseau M (2017). Vasoactive intestinal peptide dampens formyl-peptide-induced ROS production and inflammation by targeting a MAPK-p47(phox) phosphorylation pathway in monocytes. Mucosal Immunol.

[39] Paula MM, Petronilho F, Vuolo F, Ferreira GK, De Costa L, Santos GP (2015). Gold nanoparticles and/or N-acetylcysteine mediate carrageenan-induced inflammation and oxidative stress in a concentration-dependent manner. J Biomed Mater Res A.

[40] Murai N, Nagai K, Fujisawa H, Hatanaka K, Kawamura M, Harada Y (2003). Concurrent evolution and resolution in an acute inflammatory model of rat carrageenin-induced pleurisy. J Leukoc Biol.

[41] Fröde TS, Souza GE, Calixto JB (2002). The effects of IL-6 and IL-10 and their specific antibodies in the acute inflammatory responses induced by carrageenan in the mouse model of pleurisy. Cytokine.

[42] Sandes SMS, Heimfarth L, Brito RG, Santos PL, Gouveia DN, Carvalho AMS (2018). Evidence for the involvement of TNF-α, IL-1β and IL-10 in the antinociceptive and anti-inflammatory effects of indole-3-guanylhydrazone hydrochloride, an aromatic aminoguanidine, in rodents. Chem Biol Interact.

[43] Garbacki N, Tits M, Angenot L, Damas J (2004). Inhibitory effects of proanthocyanidins from Ribes nigrum leaves on carrageenin acute inflammatory reactions induced in rats. BMC Pharmacol.

[44] da Rosa JS, Facchin BM, Bastos J, Siqueira MA, Micke GA, Dalmarco EM (2013). Systemic administration of Rosmarinus officinalis attenuates the inflammatory response induced by carrageenan in the mouse model of pleurisy. Planta Med.

[45] Cuzzocrea S, Mazzon E, Dugo L, Serraino I, Di Paola R, Genovese T (2002). Absence of endogenous interleukin-10 enhances the evolution of acute lung injury. Eur Cytokine Netw.

[46] Aliberti J, Viola JP, Vieira-de-Abreu A, Bozza PT, Sher A, Scharfstein J (2003). Cutting edge: bradykinin induces IL-12 production by dendritic cells: a danger signal that drives Th1 polarization. J Immunol.

[47] Zaidan I, Tavares LP, Sugimoto MA, Lima KM, Negreiros-Lima GL, Teixeira LC et al. Angiotensin-(1–7)/MasR axis promotes migration of monocytes/macrophages with a regulatory phenotype to perform phagocytosis and efferocytosis. JCI Insight. 2022;7(1). 10.1172/jci.insight.147819 PMC8765051 34874920

[48] Nepal S, Tiruppathi C, Tsukasaki Y, Farahany J, Mittal M, Rehman J (2019). STAT6 induces expression of Gas6 in macrophages to clear apoptotic neutrophils and resolve inflammation. Proc Natl Acad Sci U S A.

[49] Zhong X, Lee HN, Kim SH, Park SA, Kim W, Cha YN (2018). Myc-nick promotes efferocytosis through M2 macrophage polarization during resolution of inflammation. FASEB J.

[50] Lucas M, Zhang X, Prasanna V, Mosser DM (2005). ERK activation following macrophage FcgammaR ligation leads to chromatin modifications at the IL-10 locus. J Immunol.

[51] Liu M, Shi P, Sumners C (2016). Direct anti-inflammatory effects of angiotensin-(1–7) on microglia. J Neurochem.

[52] Hay M, Polt R, Heien ML, Vanderah TW, Largent-Milnes TM, Rodgers K (2019). A Novel Angiotensin-(1–7) glycosylated mas receptor agonist for treating vascular cognitive impairment and inflammation-related memory dysfunction. J Pharmacol Exp Ther.

[53] Chen QF, Kuang XD, Yuan QF, Hao H, Zhang T, Huang YH (2018). Lipoxin A(4) attenuates LPS-induced acute lung injury via activation of the ACE2-Ang-(1–7)-Mas axis. Innate Immun.

[54] Fine JS, Rojas-Triana A, Jackson JV, Engstrom LW, Deno GS, Lundell DJ (2003). Impairment of leukocyte trafficking in a murine pleuritis model by IL-4 and IL-10. Inflammation.

[55] Negreiros-Lima GL, Lima KM, Moreira IZ, Jardim BLO, Vago JP, Galvão I et al. Cyclic AMP regulates key features of macrophages via PKA: recruitment, reprogramming and efferocytosis. Cells. 2020;9(1). 10.3390/cells9010128 PMC7017228 31935860

[56] da Rosa JS, Nascimento M, Parisotto EB, Lima TC, Santin JR, Biavatti MW et al. (2019) Phenolic compounds isolated from calea uniflora less. promote anti-inflammatory and antioxidant effects in mice neutrophils (ex vivo) and in mice pleurisy model (in vivo). Mediators Inflamm. 2019:1468502. 10.1155/2019/1468502 PMC6875232 31780857

[57] Zhang M, Niu YR, Liu JY, Wei XS, Wang XR, Ye LL (2019). Interleukin-26 upregulates interleukin-22 production by human CD4(+) T cells in tuberculous pleurisy. J Mol Med (Berl).

[58] Moreira-Teixeira L, Redford PS, Stavropoulos E, Ghilardi N, Maynard CL, Weaver CT (2017). T cell-derived IL-10 Impairs Host Resistance to Mycobacterium tuberculosis infection. J Immunol.

[59] Canaday DH, Wilkinson RJ, Li Q, Harding CV, Silver RF, Boom WH (2001). CD4(+) and CD8(+) T cells kill intracellular Mycobacterium tuberculosis by a perforin and Fas/Fas ligand-independent mechanism. J Immunol.

[60] Stenger S, Mazzaccaro RJ, Uyemura K, Cho S, Barnes PF, Rosat JP (1997). Differential effects of cytolytic T cell subsets on intracellular infection. Science.

[61] Stenger S, Hanson DA, Teitelbaum R, Dewan P, Niazi KR, Froelich CJ (1998). An antimicrobial activity of cytolytic T cells mediated by granulysin. Science.

[62] Barnes PF, Lu S, Abrams JS, Wang E, Yamamura M, Modlin RL (1993). Cytokine production at the site of disease in human tuberculosis. Infect Immun.

[63] Barnes PF, Chatterjee D, Abrams JS, Lu S, Wang E, Yamamura M (1992). Cytokine production induced by Mycobacterium tuberculosis Lipoarabinomannan. Relationship to chemical structure. J Immunol.

[64] Olobo JO, Geletu M, Demissie A, Eguale T, Hiwot K, Aderaye G (2001). Circulating TNF-alpha, TGF-beta, and IL-10 in tuberculosis patients and healthy contacts. Scand J Immunol.

[65] Yanagawa H, Takeuchi E, Suzuki Y, Ohmoto Y, Bando H, Sone S (1999). Presence and potent immunosuppressive role of interleukin-10 in malignant pleural effusion due to lung cancer. Cancer Lett.

[66] Aoe K, Hiraki A, Murakami T, Murakami K, Makihata K, Takao K (2003). Relative abundance and patterns of correlation among six cytokines in pleural fluid measured by cytometric bead array. Int J Mol Med.

[67] Schierloh P, Landoni V, Balboa L, Musella RM, Castagnino J, Moraña E (2014). Human pleural B-cells regulate IFN-γ production by local T-cells and NK cells in a Mycobacterium tuberculosis-induced delayed hypersensitivity reaction. Clin Sci (Lond).

[68] Murray PJ, Wang L, Onufryk C, Tepper RI, Young RA (1997). T cell-derived IL-10 antagonizes macrophage function in mycobacterial infection. J Immunol.

[69] Marshall BG, Chambers MA, Wangoo A, Shaw RJ, Young DB (1997). Production of tumor necrosis factor and nitric oxide by macrophages infected with live and dead mycobacteria and their suppression by an interleukin-10-secreting recombinant. Infect Immun.

[70] Suarez GV, Melucci Ganzarain CDC, Vecchione MB, Trifone CA, Marín Franco JL, Genoula M (2019). PD-1/PD-L1 pathway modulates macrophage susceptibility to Mycobacterium tuberculosis Specific CD8(+) T cell Induced Death. Sci Rep.

[71] de la Barrera S, Aleman M, Musella R, Schierloh P, Pasquinelli V, Garcia V (2004). IL-10 down-regulates costimulatory molecules on Mycobacterium tuberculosis-pulsed macrophages and impairs the lytic activity of CD4 and CD8 CTL in tuberculosis patients. Clin Exp Immunol.

[72] de Waal Malefyt R, Haanen J, Spits H, Roncarolo MG, te Velde A, Figdor C (1991). Interleukin 10 (IL-10) and viral IL-10 strongly reduce antigen-specific human T cell proliferation by diminishing the antigen-presenting capacity of monocytes via downregulation of class II major histocompatibility complex expression. J Exp Med.

[73] Geffner L, Basile JI, Yokobori N, Sabio YGC, Musella R, Castagnino J (2014). CD4(+) CD25(high) forkhead box protein 3(+) regulatory T lymphocytes suppress interferon-γ and CD107 expression in CD4(+) and CD8(+) T cells from tuberculous pleural effusions. Clin Exp Immunol.

[74] Qin XJ, Shi HZ, Liang QL, Huang LY, Yang HB (2008). CD4 + CD25 + regulatory T lymphocytes in tuberculous pleural effusion. Chin Med J (Engl).

[75] Sun Q, Zhang Q, Xiao H, Cui H, Su B (2012). Significance of the frequency of CD4 + CD25 + CD127- T-cells in patients with pulmonary tuberculosis and diabetes mellitus. Respirology.

[76] Ye ZJ, Yuan ML, Zhou Q, Du RH, Yang WB, Xiong XZ (2012). Differentiation and recruitment of Th9 cells stimulated by pleural mesothelial cells in human Mycobacterium tuberculosis infection. PLoS ONE.

[77] Veldhoen M, Uyttenhove C, van Snick J, Helmby H, Westendorf A, Buer J (2008). Transforming growth factor-beta ‘reprograms’ the differentiation of T helper 2 cells and promotes an interleukin 9-producing subset. Nat Immunol.

[78] Tong ZH, Shi HZ (2013). Subpopulations of helper T lymphocytes in tuberculous pleurisy. Tuberc (Edinb Scotl).

[79] Iwata Y, Matsushita T, Horikawa M, Dilillo DJ, Yanaba K, Venturi GM (2011). Characterization of a rare IL-10-competent B-cell subset in humans that parallels mouse regulatory B10 cells. Blood.

[80] Fillatreau S (2011). Novel regulatory functions for toll-like receptor-activated B cells during intracellular bacterial infection. Immunol Rev.

[81] Bouaziz JD, Calbo S, Maho-Vaillant M, Saussine A, Bagot M, Bensussan A (2010). IL-10 produced by activated human B cells regulates CD4(+) T-cell activation in vitro. Eur J Immunol.

[82] Flores-Borja F, Bosma A, Ng D, Reddy V, Ehrenstein MR, Isenberg DA (2013). CD19 + CD24hiCD38hi B cells maintain regulatory T cells while limiting TH1 and TH17 differentiation. Sci Transl Med.

[83] Chen YM, Yang WK, Whang-Peng J, Kuo BI, Perng RP (1996). Elevation of interleukin-10 levels in malignant pleural effusion. Chest.

[84] Chen YM, Yang WK, Whang-Peng J, Tsai CM, Perng RP (2001). An analysis of cytokine status in the serum and effusions of patients with tuberculous and lung cancer. Lung Cancer.

[85] Wojtowicz-Praga S (1997). Reversal of tumor-induced immunosuppression: a new approach to cancer therapy. J Immunother.

[86] Chen YM, Yang WK, Ting CC, Tsai WY, Yang DM, Whang-Peng J (1997). Cross regulation by IL-10 and IL-2/IL-12 of the helper T cells and the cytolytic activity of lymphocytes from malignant effusions of lung cancer patients. Chest.

[87] Nakamura Y, Ozaki T, Yanagawa H, Yasuoka S, Ogura T (1990). Eosinophil colony-stimulating factor induced by administration of interleukin-2 into the pleural cavity of patients with malignant pleurisy. Am J Respir Cell Mol Biol.

[88] Zenewicz LA, Yancopoulos GD, Valenzuela DM, Murphy AJ, Stevens S, Flavell RA (2008). Innate and adaptive interleukin-22 protects mice from inflammatory bowel disease. Immunity.

[89] Pestka S, Krause CD, Sarkar D, Walter MR, Shi Y, Fisher PB (2004). Interleukin-10 and related cytokines and receptors. Annu Rev Immunol.

[90] Renauld JC (2003). Class II cytokine receptors and their ligands: key antiviral and inflammatory modulators. Nat Rev Immunol.

[91] Kotenko SV, Izotova LS, Mirochnitchenko OV, Esterova E, Dickensheets H, Donnelly RP (2001). Identification of the functional interleukin-22 (IL-22) receptor complex: the IL-10R2 chain (IL-10Rbeta) is a common chain of both the IL-10 and IL-22 (IL-10-related T cell-derived inducible factor, IL-TIF) receptor complexes. J Biol Chem.

[92] Wolk K, Kunz S, Witte E, Friedrich M, Asadullah K, Sabat R (2004). IL-22 increases the innate immunity of tissues. Immunity.

[93] Sonnenberg GF, Fouser LA, Artis D (2011). Border patrol: regulation of immunity, inflammation and tissue homeostasis at barrier surfaces by IL-22. Nat Immunol.

[94] Zheng Y, Danilenko DM, Valdez P, Kasman I, Eastham-Anderson J, Wu J (2007). Interleukin-22, a T(H)17 cytokine, mediates IL-23-induced dermal inflammation and acanthosis. Nature.

[95] Wilson NJ, Boniface K, Chan JR, McKenzie BS, Blumenschein WM, Mattson JD (2007). Development, cytokine profile and function of human interleukin 17-producing helper T cells. Nat Immunol.

[96] Wolk K, Witte E, Reineke U, Witte K, Friedrich M, Sterry W (2005). Is there an interaction between interleukin-10 and interleukin-22?. Genes Immun.

[97] Zheng Y, Valdez PA, Danilenko DM, Hu Y, Sa SM, Gong Q (2008). Interleukin-22 mediates early host defense against attaching and effacing bacterial pathogens. Nat Med.

[98] Aujla SJ, Chan YR, Zheng M, Fei M, Askew DJ, Pociask DA (2008). IL-22 mediates mucosal host defense against Gram-negative bacterial pneumonia. Nat Med.

[99] Sanjabi S, Zenewicz LA, Kamanaka M, Flavell RA (2009). Anti-inflammatory and pro-inflammatory roles of TGF-beta, IL-10, and IL-22 in immunity and autoimmunity. Curr Opin Pharmacol.

[100] Qiao D, Yang BY, Li L, Ma JJ, Zhang XL, Lao SH (2011). ESAT-6- and CFP-10-specific Th1, Th22 and Th17 cells in tuberculous pleurisy may contribute to the local immune response against Mycobacterium tuberculosis infection. Scand J Immunol.

[101] Matthews K, Wilkinson KA, Kalsdorf B, Roberts T, Diacon A, Walzl G (2011). Predominance of interleukin-22 over interleukin-17 at the site of disease in human tuberculosis. Tuberc (Edinb Scotl).

[102] Brüstle A, Heink S, Huber M, Rosenplänter C, Stadelmann C, Yu P (2007). The development of inflammatory T(H)-17 cells requires interferon-regulatory factor 4. Nat Immunol.

[103] Lee WW, Kang SW, Choi J, Lee SH, Shah K, Eynon EE (2010). Regulating human Th17 cells via differential expression of IL-1 receptor. Blood.

[104] Behrends J, Renauld JC, Ehlers S, Hölscher C (2013). IL-22 is mainly produced by IFNγ-secreting cells but is dispensable for host protection against Mycobacterium tuberculosis infection. PLoS ONE.

[105] Vivier E, Spits H, Cupedo T (2009). Interleukin-22-producing innate immune cells: new players in mucosal immunity and tissue repair?. Nat Rev Immunol.

[106] Zeng G, Chen CY, Huang D, Yao S, Wang RC, Chen ZW (2011). Membrane-bound IL-22 after de novo production in tuberculosis and anti-mycobacterium tuberculosis effector function of IL-22 + CD4 + T cells. J Immunol.

[107] Li L, Qiao D, Fu X, Lao S, Zhang X, Wu C (2011). Identification of Mycobacterium tuberculosis-specific Th1, Th17 and Th22 cells using the expression of CD40L in tuberculous pleurisy. PLoS ONE.

[108] Ye ZJ, Zhou Q, Yuan ML, Du RH, Yang WB, Xiong XZ (2012). Differentiation and recruitment of IL-22-producing helper T cells stimulated by pleural mesothelial cells in tuberculous pleurisy. Am J Respir Crit Care Med.

[109] Liu Y, Ou Q, Liu Q, Gao Y, Wu J, Zhang B (2017). The expressions and roles of different forms of IL-22 in Mycobacterium tuberculosis infection. Tuberc (Edinb Scotl).

[110] Ouyang W, Kolls JK, Zheng Y (2008). The biological functions of T helper 17 cell effector cytokines in inflammation. Immunity.

[111] Volpe E, Touzot M, Servant N, Marloie-Provost MA, Hupé P, Barillot E (2009). Multiparametric analysis of cytokine-driven human Th17 differentiation reveals a differential regulation of IL-17 and IL-22 production. Blood.

[112] Ye Z, Li M, Mei Z, Zhen G, Zhang P (2015). [The effects and mechanisms of interleukin-22 and interferon-γ on epithelial-mesenchymal transition of pleural mesothelial cells]. Zhonghua Jie He He Hu Xi Za Zhi.

[113] Sallusto F, Lenig D, Förster R, Lipp M, Lanzavecchia A (1999). Two subsets of memory T lymphocytes with distinct homing potentials and effector functions. Nature.

[114] Fickenscher H, Pirzer H (2004). Interleukin-26. Int Immunopharmacol.

[115] Sheikh F, Baurin VV, Lewis-Antes A, Shah NK, Smirnov SV, Anantha S (2004). Cutting edge: IL-26 signals through a novel receptor complex composed of IL-20 receptor 1 and IL-10 receptor 2. J Immunol.

[116] Wolk K, Kunz S, Asadullah K, Sabat R (2002). Cutting edge: immune cells as sources and targets of the IL-10 family members?. J Immunol.

[117] Donnelly RP, Sheikh F, Dickensheets H, Savan R, Young HA, Walter MR (2010). Interleukin-26: an IL-10-related cytokine produced by Th17 cells. Cytokine Growth Factor Rev.

[118] Pène J, Chevalier S, Preisser L, Vénéreau E, Guilleux MH, Ghannam S (2008). Chronically inflamed human tissues are infiltrated by highly differentiated Th17 lymphocytes. J Immunol.

[119] Tengvall S, Che KF, Lindén A (2016). Interleukin-26: an emerging player in Host Defense and inflammation. J Innate Immun.

[120] Meller S, Di Domizio J, Voo KS, Friedrich HC, Chamilos G, Ganguly D (2015). T(H)17 cells promote microbial killing and innate immune sensing of DNA via interleukin 26. Nat Immunol.

[121] Che KF, Tengvall S, Levänen B, Silverpil E, Smith ME, Awad M (2014). Interleukin-26 in antibacterial host defense of human lungs. Effects on neutrophil mobilization. Am J Respir Crit Care Med.

[122] Dambacher J, Beigel F, Zitzmann K, De Toni EN, Göke B, Diepolder HM (2009). The role of the novel Th17 cytokine IL-26 in intestinal inflammation. Gut.

[123] Corvaisier M, Delneste Y, Jeanvoine H, Preisser L, Blanchard S, Garo E (2012). IL-26 is overexpressed in rheumatoid arthritis and induces proinflammatory cytokine production and Th17 cell generation. PLoS Biol.

[124] Kaabachi W, Bouali E, Berraïes A, Dhifallh IB, Hamdi B, Hamzaoui K (2017). Interleukin-26 is overexpressed in Behçet’s disease and enhances Th17 related -cytokines. Immunol Lett.

[125] Guerra-Laso JM, Raposo-García S, García-García S, Diez-Tascón C, Rivero-Lezcano OM (2015). Microarray analysis of Mycobacterium tuberculosis-infected monocytes reveals IL26 as a new candidate gene for tuberculosis susceptibility. Immunology.

